# Dietary ketosis improves circadian dysfunction as well as motor symptoms in the BACHD mouse model of Huntington’s disease

**DOI:** 10.3389/fnut.2022.1034743

**Published:** 2022-11-03

**Authors:** Daniel S. Whittaker, T. Katherine Tamai, Raj S. Bains, Sophia Anne Marie Villanueva, Shu Hon Christopher Luk, Derek Dell’Angelica, Gene D. Block, Cristina A. Ghiani, Christopher S. Colwell

**Affiliations:** ^1^Department of Psychiatry and Biobehavioral Sciences, David Geffen School of Medicine, University of California, Los Angeles, Los Angeles, CA, United States; ^2^Department of Pathology and Laboratory Medicine, David Geffen School of Medicine, University of California, Los Angeles, Los Angeles, CA, United States

**Keywords:** BACHD mice, circadian rhythms, ketogenic diet (KD), sleep, motor performance, activity rhythm

## Abstract

Disturbances in sleep/wake cycles are common among patients with neurodegenerative diseases including Huntington’s disease (HD) and represent an appealing target for chrono-nutrition-based interventions. In the present work, we sought to determine whether a low-carbohydrate, high-fat diet would ameliorate the symptoms and delay disease progression in the BACHD mouse model of HD. Adult WT and BACHD male mice were fed a normal or a ketogenic diet (KD) for 3 months. The KD evoked a robust rhythm in serum levels of β-hydroxybutyrate and dramatic changes in the microbiome of male WT and BACHD mice. NanoString analysis revealed transcriptional changes driven by the KD in the striatum of both WT and BACHD mice. Disturbances in sleep/wake cycles have been reported in mouse models of HD and are common among HD patients. Having established that the KD had effects on both the WT and mutant mice, we examined its impact on sleep/wake cycles. KD increased daytime sleep and improved the timing of sleep onset, while other sleep parameters were not altered. In addition, KD improved activity rhythms, including rhythmic power, and reduced inappropriate daytime activity and onset variability. Importantly, KD improved motor performance on the rotarod and challenging beam tests. It is worth emphasizing that HD is a genetically caused disease with no known cure. Life-style changes that not only improve the quality of life but also delay disease progression for HD patients are greatly needed. Our study demonstrates the therapeutic potential of diet-based treatment strategies in a pre-clinical model of HD.

## Introduction

Huntington’s disease (HD) is a progressive degenerative disorder that results in cognitive, psychiatric and motor dysfunction (1, 2). HD is caused by a CAG repeat expansion within the huntingtin (*HTT*) gene, which encodes an enlarged polyglutamine tract in the N-terminal fragment of the protein, causing mutant huntingtin to fold abnormally (3). The resulting protein misfolding and aggregations lead to pathophysiology and cell death in the brain (4) but also impacts function throughout the body (5). Among the diverse set of HD symptoms, altered central and peripheral metabolism of glucose, lipids and carbohydrates has been well described in HD patients (6, 7). In addition, sleep disorders are common among patients and animal models recapitulate the progressive breakdown of the circadian rest/activity cycle seen in the patients including delays in sleep onset, sleep fragmentation, and an increase in cycle-to-cycle variability (8–10). Given the many links between sleep/wake cycles and metabolism (11, 12), these symptoms could be co-dependent. Hence, HD represents a promising target for chrono-nutrition-based interventions.

Among the most developed of the chronotherapies is time-restricted feeding [TRF; (13)] for which there is a detailed biochemical understanding in both liver (14, 15) and pancreas (16). In addition, there is an emerging literature on clinical trials using TRF protocols (17–20). Over 90 clinical trials labeled with the key word “time-restricted feeding” are presently listed on the ClinicalTrials.gov online database (as of September, 2022). In preclinical mouse models of HD, a TRF protocol (6 h feeding aligned to the middle of the active phase and 18 h fasting) has been shown to effectively improve circadian parameters, autonomic functions as well as motor performance (21, 22). An important consequence of TRF is an increase in ketone bodies (23) and there has been speculation that at least some of the benefits of TRF are due to the switch from glucose to lipids as fuel source and the subsequent reduction in reactive oxygen species. This raises the question of whether a ketogenic diet (KD), similar to TRF, could also be beneficial for HD and other neurodegenerative disorders (24–26).

Therefore, in the current study, we first sought to demonstrate that KD is biologically impactful, both peripherally and centrally, in the BACHD mouse model of HD. The possible effects of the diet were first evaluated on body weight, serum levels of β-hydroxybutyrate (βHB), one of the most abundant ketone bodies, species composition of fecal microbiome and gene expression in the striatum in wild-type (WT) and mutant mice. Then, we examined the impact of the KD on sleep behavior, activity rhythms and motor performance (rotarod, grip strength, challenging beam).

## Materials and methods

The work presented in this study followed all the guidelines and regulations of the UCLA Division of Animal Medicine that are consistent with the Animal Welfare Policy Statements and the recommendations of the Panel on Euthanasia of the American Veterinary Medical Association.

### Animals

The BACHD mouse model used in this study expresses the full-length of the human mutant HTT gene encoding 97 glutamine repeats under the control of the endogenous regulatory machinery (27). BACHD dams, backcrossed on the C57BL/6J background for a minimum of 12 generations, were bred with C57BL/6J (WT) males from The Jackson Laboratory (Bar Harbor, Maine) in our facility at UCLA. Data were collected from male WT and heterozygous for the BACHD transgene littermates. Genotyping was performed at 15 days of age by tail snips, and after weaning at postnatal day 21, littermates were group housed, unless otherwise noted. All animals were housed in soundproof chambers with controlled temperature, humidity and lighting conditions, 12 h light, 12 h dark cycle (12:12 LD, intensity 350 lux) for at least 2-weeks prior to any experimentation or change in diet. For all experiments, a light meter (BK precision, Yorba Linda, CA) was used to measure light-intensity (lux). Each chamber holds 8 cages of mice, grouped together by feeding treatment. The animals received cotton nestlets and had water available at all times.

### Experimental groups and diet

Male WT and BACHD mice (3 months old) were randomly assigned to either a Normal Diet (ND) or KD group. The mice had *ad libitum* access to either a custom KD (Teklad diet TD.10911.PWD, Envigo, Madison, WI) or ND (Teklad diet 7013, NIH-31 Modified Open Formula diet, Envigo) for 3 months. The KD used (77.1% fat, 22.4% protein, 0.5% carbohydrate) has moderately high protein, no sugars and predominantly healthy fats (with a 2:1 ratio of n-3 to n-6 fatty acids from medium chain triglycerides with a little flax and canola oil). The level of proteins in the KD diet is consistent with recommendations for optimal health. The food was refreshed every 4 days, new and unconsumed food were weighed. At the end of the treatment, the animals (6–7 months old) were used to assess motor functions and then euthanized for tissue collection.

### Body weight and body composition

For the first 3 weeks, to ensure that the KD did not hinder the animals’ normal growth, WT and BACHD mice (*n* = 8 animals/genotype) on either ND or KD were weighed on the same day the food was refreshed (every 4 days), then only once every 4 weeks, and again at the end of the treatment period. The animals were weighed in the middle of the LD cycle between Zeitgeber time (ZT) 10 and ZT12. The body weights recorded at the end of the treatment period are shown.

Body composition (*n* = 4 animals/genotype/diet regimen) was determined at about 6+ months of age with a Mouse Minispec apparatus (Bruker Woodlands, TX) with Echo Medical Systems (Houston, TX) software. This apparatus uses NMR spectroscopy for fat and lean mass measurements with coefficients of variation of < 3%. Correlation between NMR and gravimetric measurements is better than 0.99.

### β-hydroxybutyrate measurements

Tail vein blood samples were obtained from 6 months old WT and BACHD mice (*n* = 10 animals/genotype) kept for 3 months on a ND or KD at 6 time points during the sleep/wake cycle (ZT 2, 6, 10, 14, 18, 22). A small incision was made to access the tail vein to permit repeated withdrawals (under 3 μL per collection) with minimal pain and distress to the mice. At each specific ZT, mice were retrieved from the cages, placed on a stable surface and minimally restrained by the tail. Blood sampling was performed under normal room lighting (350 lux) for the testing times in the day, between ZT 0 and 12, and under dim red-light (3 lux) for those during the night (between ZT12 and 24). Blood flow was stopped by applying pressure with a sterile gauze to achieve hemostasis. Metabolite measurements were made in 1.5 μL blood samples using a commercially available ketone meter (Precision Xtra Ketone Monitoring System, Abbott Laboratories, Chicago, IL).

### Microbiome measurements

Fecal samples were collected at ZT6 from WT and BACHD mice (*n* = 5 animals/genotype) that had been on ND or KD for at least 3 months. The fecal samples were then sent to TransnetYX (TransnetYXyx, Inc., Cordova, TN) for sequencing of the gut microbiome. The composition of the gut microbiome and the species relative abundances were analyzed using the One Codex (One Codex, San Francisco, CA) platform.

### RNA extraction and nanostring analysis

WT and BACHD (6–7 months old) mice (*n* = 5–6 animals/genotype) on either ND or KD were euthanized with isoflurane at ZT 14. The left and right striati were rapidly dissected out, separately frozen and stored at -80°C. Samples were lysed using the Invitrogen™ TRIzol™ reagent (Thermo Fisher Scientific; Carlsbad, CA). Total RNA was extracted using the RNeasy^^®^^ Mini kit (Qiagen). Concentration and purity of the samples were assessed using a ThermoScientific™ NanoDrop™ One Microvolume UV-Vis Spectrophotometer (Canoga Park, CA). Gene analysis was performed in 150 ng of total RNA (at a concentration of 20 ng/μl) at the UCLA Center for Systems Biomedicine (genetic engineering platform) using the Nanostring nCounter^^®^^ Neuropathology Panel designed to interrogate 770 transcripts specific for neurodegenerative processes/disease. Data quality and normalization of the sample signals were performed using the nSolver analyses software. The Rosalind^^®^^ software^1^ was used to identify differentially expressed genes, as well as to obtain fold changes and *p*-values within two groups comparison (4 combinations) with genotype and diet as attributes as described in the ROSALIND^^®^^ Nanostring Gene Expression Methods. The Reactome database was used to identify the top enriched biological pathways (Rosalind interactive analysis).^2^ The identified genes along with selected genes known to be circadian regulated were further analyzed by two-way ANOVA followed by the Holm-Sidak’s multiple comparisons test using the relative expression values obtained with the nSolver software.

### Locomotor activity rhythms

WT and BACHD mice on ND or KD (*n* = 8 animals/genotype) were singly housed in cages with IR motion sensors to record their locomotor activity with a 12:12 h LD cycle and entrained for 2 weeks before beginning data collection. Activity data were collected in the 2–3 weeks prior to the motor performance tests when the mice were between 5 and 6 months of age and analyzed using the El Temps (A. Diez-Nogura, Barcelona, Spain)^3^ and ClockLab programs. Locomotor activity was recorded as previously described (28) using Mini Mitter (Bend, OR) data loggers in 3-min bins, and 7–10 days of data were averaged for analysis. The data were analyzed to determine the period and rhythmic strength as previously described (28). The periodogram analysis uses a χ^2^-test with a threshold of 0.001 significance, from which the amplitude of the periodicities is determined at the circadian harmonic to obtain the rhythm power. The amount of cage activity over a 24 h period was averaged and reported here as the arbitrary units (a.u.)/h. The number of activity bouts and the average length of bouts were determined using Clocklab, where each bout was counted when activity bouts were separated by a gap of 21 min (maximum gap: 21 min; threshold: 3 counts/min). The onset variability was determined using Clocklab by drawing the best-fit line over the recorded days and averaging the differences between activity onset and best-fit regression of each day.

A separate cohort of WT and BACHD mice on ND or KD was held in LD for about 2 months followed by 4 weeks in constant darkness (DD). Cage activity was recorded to obtain free-running activity.

### Sleep behavior

Immobility-defined sleep was determined as described previously (28) in 6–7 months old WT and BACHD mice (*n* = 8 animals/genotype) held for 3+ months on ND or KD. Animals were housed in see-through plastic cages containing bedding (without the addition of nesting material). A side-on view of each cage was obtained, with minimal occlusion by the food bin or water bottle, both of which were top-mounted. Cages were top-lit using IR LED lights. Video capture was accomplished using surveillance cameras with visible light filters (Gadspot Inc., City of Industry, CA) connected to a video-capture card (Adlink Technology Inc., Irvine, CA) on a custom-built computer system. ANY-maze software (Stoelting Co., Wood Dale, IL) was used for automated tracking of mouse immobility.

Immobility was registered when 95% of the area of the animal stayed immobile for more than 40 s, as was previously determined to have 99% correlation with simultaneous EEG/EMG defined sleep (29). Continuous tracking of the mice was performed for a minimum of 5 sleep-wake cycles, with randomized visits (1–2 times/day) by the experimenter to confirm mouse health and video recording. The 3rd and 4th sleep-wake cycles were averaged for further analysis. Immobility-defined sleep data were exported in 1 min bins, and total sleep time was determined by summing the immobility durations in the rest phase (ZT 0–12) or active phase (ZT 12–24). An average waveform of hourly immobile-sleep over the two sleep-wake cycles was produced per genotype and treatment for graphical display. Variability of sleep onset, sleep offset, and sleep fragmentation were determined using Clocklab (Actimetrics, Wilmette, IL).

### Motor behavior

All the motor behavioral tests were performed in WT and BACHD mice fed with either ND or KD at about 6.5–7 months of age (*n* = 8–10 mice/genotype) after monitoring sleep and activity rhythms.

#### Grip strength test

Grip strength was used to measure neuromuscular function as maximal muscle strength of forelimbs. The grip strength ergometer (Santa Cruz Biotechnology, Santa Cruz, CA) was set up on a flat surface with a mouse grid firmly secured in place. The grid was cleaned with 70% ethanol and allowed to dry before testing each cohort. Peak mode was selected to enable measurement of maximal strength exerted. The sensor is reset to zero before each trial. Well-handled mice were tested in their active phase under dim red light (3 Lux) and acclimated to the testing room for 10 min prior to testing. Mice underwent five trials with an inter-trial interval of at least 2 min. For each trial, each mouse was removed from its home cage by gripping the tail between the thumb and the forefinger. The mice were lowered slowly over the grid, and only their forepaws were allowed to grip the grid. Mice were pulled by the tail ensuring the torso remains horizontal until they were no longer able to grip the grid. Mice were then returned to their cages. The maximal grip strength value of each mouse was utilized.

#### Rotarod test—Accelerating version

The rotarod apparatus (Ugo Basile, Varese, Italy) is commonly used to measure motor coordination and balance. This apparatus is, in essence, a small circular treadmill. It consists of an axle or rod thick enough for a mouse to stand over the top of when it is not in motion and a flat platform a short distance below the rod. The rod was covered with smooth rubber to provide traction while preventing the mice from clinging to the rod. In this study, mice were placed on top of the rubber-covered rod. When the mice moved at the pace set by the rotation rate of the rod, they would stay on top of it. When mice no longer moved at the selected pace, they dropped a short distance to the platform below. The time a mouse remains on the rod, before dropping to the platform is the latency to fall. Following a 15-min habituation to the testing room, mice were placed on the slowly rotating rod. The rod gradually accelerates from 5 to 38 rpm over the course of the trial. The length of time the mouse stayed on the rod was recorded. A 2-day protocol for the accelerating rotarod tests was used. On the first day, the mice were trained on the rotarod over 5 trials. The maximum length of each trial was 600 s, and mice were allowed to rest for a minimum of 60 s between trials. On the second day, mice were tested on the rotarod and the latency to fall from the rotarod was recorded from 5 trials. Mice were again allowed to rest for a minimum of 60 s between trials. Data from each mouse were analyzed after averaging the times from all five trials. The apparatus was cleaned with 70% alcohol and allowed to dry completely between trials. A dim red-light (3 lux) was used for illumination during active (dark) phase testing.

#### Challenging beam test

In our version of the test, a beam was placed between two cages. The beam narrows in 4 intervals from 33 mm > 24 mm > 18 mm > 6 mm, with each segment spanning 253 mm in length. The home cage of each mouse was put on the end of the beam as the motivating factor. In this study, animals were trained on the beam for 5 consecutive trials on two consecutive days. During each trial, each mouse was placed on the widest end of the beam and allowed to cross with minimal handling by the experimenter. On the testing day, a metal grid (10 × 10 mm spacing, formed using 19-gauge wire) was overlaid on the beam. This overlaid grid increased the difficulty of the beam traversal task and provided a visual reference for foot slips made while crossing the grid. Each mouse was evaluated on five consecutive trials conducted during their active (dark) phase. Trials were recorded by a camcorder under dim red-light conditions (2 lux), supplemented with infrared lighting for video recording. The videos were scored *post hoc* by two independent observers for the number of missteps (errors) made by each mouse. The observers were masked as to the treatment group of the mice that they were scoring. An error was scored when any foot dipped below the grid. The number of errors was averaged across the 5 trials per mouse to give the final reported values. The apparatus was cleaned with 70% alcohol and allowed to dry completely between trials.

### Statistical analysis

The sample size per group was determined by both our empirical experience with the variability in the prior measures in the BACHD mice and a power analysis (SigmaPlot, SYSTAT Software, San Jose, CA) that assumed a power of 0.8 and an alpha of 0.05. Data sets were examined for normality (Shapiro-Wilk test) and equal variance (Brown-Forsythe test). To determine the impact of the diet on temporal activity, sleep, and ketone waveforms, we used a two-way analysis of variance (two-way ANOVA) with treatment and time as factors. All the other data were analyzed using two-way ANOVA with diet and genotype as factors. Pairwise Multiple Comparison Procedures were made using the Holm-Sidak’s method. Between-group differences were determined significant if *p* < 0.05. Values are reported in the tables as mean ± standard deviation (SD) or in the figures as mean ± standard error of the mean (SEM).

## Results

**FIGURE 1:**
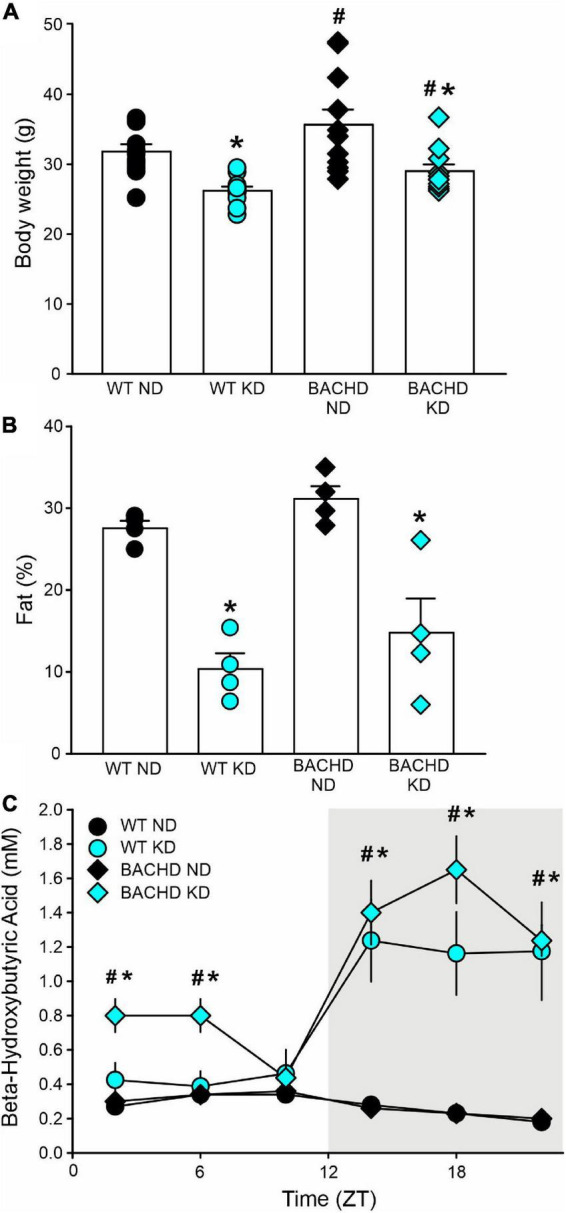
The ketogenic diet (KD) is effective in the BACHD mouse model of HD. Male WT and BACHD mice were placed on a KD at 3 months of age and held on this diet for 3 months. Controls of each genotype were kept on their normal diet (ND). **(A)** Body weight measurements at the end of the study indicated that the BACHD on ND were heavier than the WT, while the KD reduced the body weight in both genotypes (*n* = 8 mice per group). Two-way ANOVA indicated significant effects of both genotype [*F*_(1_, _43)_ = 6.241, *P* = 0.017] and diet [*F*_(1_, _43)_ = 21.128, *P* < 0.001]. **(B)** The adiposity of separate cohorts of mice (n = 4 per group) were evaluated using NMR spectroscopy. Both groups of mice on the KD were leaner as indicated by the lower% of fat in the body composition. Two-way ANOVA indicated no effect of genotype [*F*_(1_, _15)_ = 2.631, *P* = 0.131] but an unquestionable effect of the diet regimen [*F*_(1_, _15)_ = 46.0538, *P* < 0.001]. **(C)** To ensure that the BACHD mice fed the KD would undergo ketosis, tail blood was sampled at 6 time-points throughout the 24-h cycle (ZT 2, 6, 10, 14, 18, 22) and βHB measured (*n* = 10 per group). Both genotypes exhibited robust daily rhythms in ketones that peaked in the night. The BACHD mice also presented with elevated levels of βHB at some phases during the day. Two-way ANOVA found significant effects of time [WT: *F*_(5_, _107)_ = 8.833, *P* < 0.001; BAHCD: *F*_(5_, _107)_ = 20.443, *P* < 0.001] and diet [WT: *F*_(1_, _107)_ = 16.267, *P* < 0.001; BACHD: *F*_(1_, _107)_ = 76.599, *P* < 0.001]. Data are presented as means ± S.E.M. values. Significant differences were calculated using a two-way ANOVA followed by Holm–Sidak’s multiple comparisons tests: *P < 0.05 vs. mice on ND (effect of diet); ^#^*P* < 0.05 between genotypes (same diet).

In these experiments, we examined the effect(s) of a KD on BACHD and WT mice in comparison with mice on ND. Both KD and ND groups had *ad libitum* access to food from 3 to 6–7 months of age. By the end of the study, the body weights of both WT and BACHD mice held on KD were reduced as compared to their counterparts fed a ND (Figure 1A), with significant effects of both genotype and diet. Body composition analysis carried out on a separate cohort of mice showed that adiposity was higher in the BACHD and WT mice under the ND compared to those on the KD (Figure 1B), with no effect of genotype but an unquestionable effect of the diet regimen. Thus, mice on KD were leaner independent of their genotype.

Since HD patients present with metabolic deficits, to ensure that the BACHD mice would undergo ketosis in response to the KD, we sampled tail blood at 6 time-points throughout the 24-h cycle and measured βHB. Both WT and mutant mice exhibited pronounced rhythms in βHB under KD but not under control feeding conditions (Figure 1C). The βHB levels in the WT and BACHD on KD exhibited significant effects of time and diet. Interestingly, the βHB levels were significantly elevated in the mutants at most phases (ZT 2, 6, 14, 18, and 22) of the daily cycle, whilst in WT mice, the increase was only observed during the dark phase (ZT 14, 18, 22). Therefore, both genotypes responded to the KD with the rhythmic production of ketone bodies, albeit more pronounced in the BACHD mice.

**FIGURE 2:**
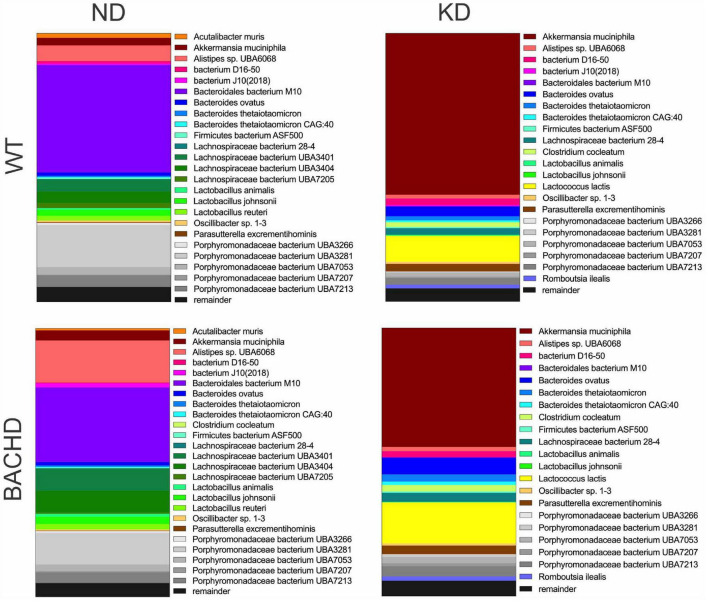
Ketogenic diet drove large changes in the microbiome of the BACHD model of HD. Male WT and BACHD mice (3 months old) were fed a normal diet (ND) or a KD for 3 months. For all four groups, fecal samples were collected at ZT6 when the mice were 6 months of age (*n* = 5 mice per group). The species abundance determined from each animal was averaged to produce a picture of the species composition for each of the four groups. The top 25 bacterial species across all 4 groups were identified and each is indicated by a different color. The probiotic *Akkermansia municiphila* (shown in brick red) dramatically increased in abundance under KD. See Table 1 for values analyzed by two-way ANOVA with genotype and diet as factors, followed by Holm-Sidak’s multiple comparisons test.

The gut microbiota is a very dynamic entity influenced by environmental and nutritional factors, which, in prior work has reported to be altered by KD (30, 31). The possible effect of KD on species composition of the gut microbiota in both genotypes was examined by sequencing fecal samples collected from WT and BACHD on both diets for 3 months. KD elicited dramatic changes in the relative abundances of the bacteria species present in the fecal samples in both genotypes (Figure 2). The top 15 species in each of the four groups (as determined by relative abundances) were compiled, and the overlapping species identified (Table 1). Most of the species displayed statistically significant differences in relative abundances due to the diet, with no effects of genotype. It is noteworthy that the probiotic *Akkermansia municiphila* dramatically increased in abundance in both KD groups. Under ND, this bacterium represented 2.8 and 3.8% of the total species found in WT and BACHD, respectively, while, under KD, this species increased to 60.2% in WT and 44% in the mutants. Both diet and the genotype had a significant effect on the abundance of one species (*Alistipes* sp. UBA6068) (Table 1) along with a significant interaction of the two factors [*F*_(1, 19)_ = 5.928; *P* = 0.027]. The evidence that effective changes were driven in the microbiome by the KD suggests that the diet is actively influencing both biological and metabolical pathways in these mutants.

**TABLE 1:** Species abundance of microbiome measured in fecal samples from WT and BACHD mice fed a normal (ND) or a ketogenic diet (KD).

	WT		BACHD			
Species	ND	KD	ND	KD	Diet	Genotype
*Akkermansia muciniphila*	2.8 ± 3.6	60.2 ± 22.9*	3.8 ± 3.9	44.4 ± 30.2*	***F*_(1, 19)_ = 32.756; *P* < 0.001**	*F*_(1, 19)_ = 0.757; *P* = 0.397
*Bacteroidales bacterium M10*	40.1 ± 13.9	0.2 ± 0.2*	27.8 ± 6.8	0.2 ± 0.3*	***F*_(1, 19)_ = 95.124; *P* < 0.001**	*F*_(1, 19)_ = 3.145; *P* = 0.095
*Alistipes* sp. *UBA6068*	5.8 ± 1.9	1.4 ± 0.6	**15.7 ± 8.5^#^**	1.6 ± 0.3*	***F*_(1, 19)_ = 22.080; P < 0.001**	***F*_(1, 19)_ = 6.450; *P* = 0.022**
*Bacteroides ovatus*	1.2 ± 0.3	3.6 ± 3.5	1.2 ± 1.1	6.2 ± 4.3*	***F*_(1, 19)_ = 8.472; *P* = 0.010**	*F*_(1, 19)_ = 1.091; *P* = 0.312
*Parasutterella excrementihominis*	0.3 ± 0.3	2.8 ± 3.8	0.2 ± 0.1	3.3 ± 3.5	***F*_(1, 19)_ = 5.977; *P* = 0.026**	*F*_(1, 19)_ = 0.016; *P* = 0.901
*Lachnospiraceae bacterium 28-4*	0.2 ± 0.2	2.5 ± 2.5	0.1 ± 0.1	3.3 ± 3.1*	***F*_(1, 19)_ = 9.744; *P* = 0.007**	*F*_(1, 19)_ = 0.190; *P* = 0.669
*bacterium D16-50*	0.9 ± 1.6	2.4 ± 2.5	0.1 ± 0.1	3.3 ± 31	*F*_(1, 19)_ = 3.904; *P* = 0.066	*F*_(1, 19)_ = 0.237; *P* = 0.633
*Bacteroides thetaiotaomicron*	0.5 ± 0.1	1.5 ± 1.5	0.5 ± 0.5	2.7 ± 1.9*	***F*_(1, 19)_ = 8.368; *P* = 0.011**	*F*_(1, 19)_ = 0.290; *P* = 0.290
*Porphyromonadaceae bacterium UBA7213*	4.4 ± 1.0	2.5 ± 1.6	4.1 ± 1.0	3.8 ± 3.4	*F*_(1, 19)_ = 1.336; *P* = 0.265	*F*_(1, 19)_ = 0.293; *P* = 0.596
*Porphyromonadaceae bacterium UBA7053*	2.8 ± 0.7	1.5 ± 1.0	2.5 ± 0.6	2.3 ± 1.9	*F*_(1, 19)_ = 1.336; *P* = 0.265	*F*_(1, 19)_ = 0.293; *P* = 0.596
*Lactobacillus reuteri*	1.7 ± 2.7	0.0 ± 0.0	2.5 ± 0.6	2.3 ± 1.9	*F*_(1, 19)_ = 4.407; *P* = 0.052	*F*_(1, 19)_ = 0.037; *P* = 0.849
*Lactobacillus johnsonii*	2.3 ± 3.0	0.1 ± 0.1	2.6 ± 3.3	0.1 ± 0.1	***F*_(1, 19)_ = 5.674; *P* = 0.030**	*F*_(1, 19)_ = 0.035; *P* = 0.853
*Porphyromonadaceae bacterium UBA3281*	15.6 ± 6.2	0.4 ± 0.6*	11.8 ± 3.8	0.9 ± 2.0*	***F*_(1, 19)_ = 59.532; *P* < 0.006**	*F*_(1, 19)_ = 1.003; *P* = 0.332
*Lachnospiraceae bacterium UBA3401*	4.4 ± 9.8	0.0 ± 0.0	8.3 ± 6.3	0.0 ± 0.0*	***F*_(1, 19)_ = 5.953; *P* = 0.027**	*F*_(1, 19)_ = 0.552; *P* = 0.468
*Lachnospiraceae bacterium UBA3404*	4.3 ± 9.0	0.0 ± 0.0	8.1 ± 6.3	0.0 ± 0.0*	***F*_(1, 19)_ = 5.812; *P* = 0.028**	*F*_(1, 19)_ = 0.532; *P* = 0.476
*Lachnospiraceae bacterium UBA7205*	1.9 ± 9.6	0.0 ± 0.0	0.5 ± 6.3	0.0 ± 0.0	***F*_(1, 19)_ = 7.375; *P* = 0.015**	*F*_(1, 19)_ = *2.494*; *P* = 0.134
*Acutalibacter muris*	1.6 ± 1.9	0.0 ± 0.0*	0.7 ± 0.5	0.0 ± 0.0	***F*_(1, 19)_ = 8.266; *P* = 0.011**	*F*_(1, 19)_ = *1.213*; *P* = 0.287
*Porphyromonadaceae bacterium UBA3266*	0.8 ± 1.8	0.1 ± 0.1*	0.7 ± 0.5	0.1 ± 0.0*	***F*_(1, 19)_ = 58.352; *P* < 0.001**	*F*_(1, 19)_ = *0.145*; *P* = 0.709
*Lactococcus lactis*	0.0 ± 0.1	9.7 ± 0.1*	0.0 ± 0.3	15.3 ± 0.2*	***F*_(1, 19)_ = 26.198; *P* < 0.001**	*F*_(1, 19)_ = 1.325; *P* = 0.267
*Clostridium cocleatum*	0.0 ± 0.0	1.7 ± 6.5*	0.0 ± 0.2	2.4 ± 8.7*	***F*_(1, 19)_ = 41.479; *P* < 0.001**	*F*_(1, 19)_ = 1.066; *P* = 0.317
*Romboutsia ilealis*	0.0 ± 0.0	1.4 ± 1.3	0.0 ± 0.0	1.5 ± 0.5*	***F*_(1, 19)_ = 9.192; *P* = 0.008**	*F*_(1, 19)_ = 0.036; *P* = 0.852
*Oscillibacter* sp. *1-3*	0.6 ± 0.4	0.8 ± 0.4	0.5 ± 02	0.8 ± 0.3	*F*_(1, 19)_ = 2.610; *P* = 0.126	*F*_(1, 19)_ = 0.217; *P* = 0.647
*Bacteroides thetaiotaomicron CAG:40*	0.2 ± 0.1	0.7 ± 0.7	0.2 ± 0.2	1.2 ± 0.9*	***F*_(1, 19)_ = 8.137; *P* = 0.012**	*F*_(1, 19)_ = 1.173; *P* = 0.295
*Firmicutes bacterium ASF500*	0.5 ± 0.5	0.5 ± 0.3	0.2 ± 0.0	0.5 ± 0.2	*F*_(1, 19)_ = 1.1280 *P* = 0.275	*F*_(1, 19)_ = 0.692; *P* = 0.418
*bacterium J10*(2018)	0.5 ± 1.1	0.3 ± 0.8	1.5 ± 2.2	0.0 ± 0.0	*F*_(1, 19)_ = 2.159; *P* = 0.161	*F*_(1, 19)_ = 0.399; *P* = 0.537

Two-way ANOVA followed by Holm-Sidak’s multiple comparisons test of the abundance of the 25 most common species (Figure 2). Data are expressed as the percentage of the total number of species in each sample and are the mean ± SD of 5 animals/genotype/diet regimen. Degrees of freedom are reported within parentheses, alpha = 0.05. Asterisks indicate significant difference within genotype (i.e., diet effect); crosshatches indicate differences between genotypes (i.e., same diet). There were no significant interactions except for *Alistipes* (see text for values). Bold type indicates statistical significance.

**FIGURE 3:**
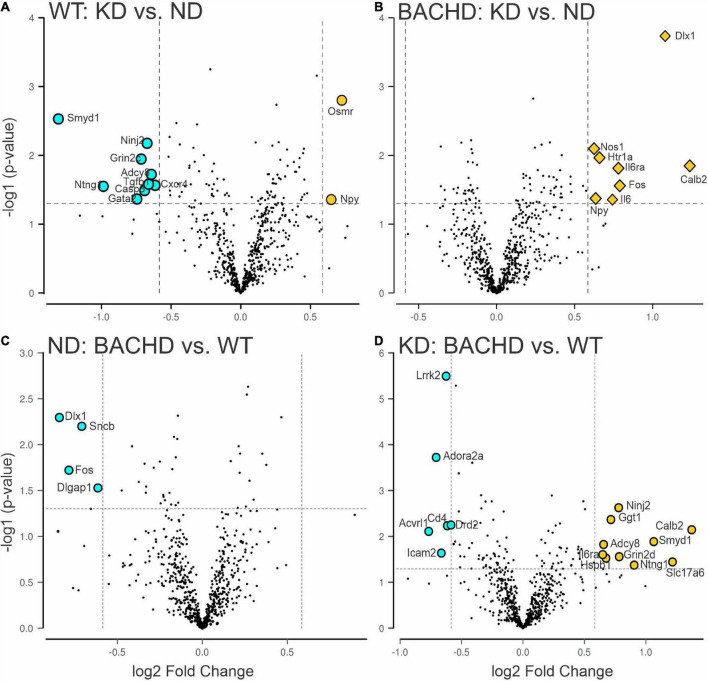
Changes in the transcriptional landscape of the BACHD striatum driven by the Ketogenic diet (KD). Striatal tissue samples were collected at ZT 14 from 6 to 7 months old WT and BACHD male mice fed a normal diet (ND) or a KD for 3+ months until euthanized (*n* = 5–6 animals per group). NanoString transcriptomic analysis was performed using the Nanostring nCounter^^®^^ Neuropathology Panel and analyzed using the software rosalind. The volcano plots illustrate the differentially expressed transcripts and similarities between WT and BACHD on ND or KD (two-groups comparisons). Volcano plot in **(A,B)** were obtained by comparing the effects of the KD within each genotype, while the plots in **(C,D)** illustrate the differences in gene expression in animals on the same diet. The gray points indicate genes with no significant changes, while significantly increased genes are in dark yellow and those decreased are shown in cyan. See Table 2 and Supplementary Figure 2 for expression levels analyzed by two-way ANOVA with genotype and diet as factors followed by Holm-Sidak’s multiple comparisons test.

One of the key pathological features of HD are transcriptional changes in the striatum (32, 33). Hence, we sought to determine if KD altered gene expression patterns in the striatum using NanoString transcriptomic analysis. A total of 30 differentially expressed genes was identified in 2 groups-comparisons (effect of diet within genotype or between genotypes), with the highest number in the BACHD-KD vs. WT-KD comparison (Figure 3 and Supplementary Figure 1). Eight genes were found to be upregulated in the BACHD-KD compared to BACHD on ND (Figure 3 and Supplementary Figure 1), while KD elicited changes in 11 genes in WT, with 2 genes upregulated and 9 downregulated. Among these, only one gene was found to be altered in both genotypes, *Npy*. When comparing the effect of the KD in BACHD and WT, there were 16 genes differentially expressed (10 upregulated and 6 downregulated), with two of the upregulated genes, *Calb2* and *Il6ra*, showing a similar change to that observed in the BACHD-KD vs. BACHD ND comparison (Figure 3 and Supplementary Figure 1). There were only 4 genes differentially expressed between the BACHD and WT mice on ND, all downregulated in the BACHD. It is notable that 2 of them, *Dlx1* and *Fos*, were upregulated in the BACHD by the KD, suggesting that this diet may influence cell function in the striatum.

Further analyses of the 30 identified genes using two-way ANOVA with diet and genotype as factors (Table 2 and Supplementary Figure 2) revealed that among those significantly affected by the diet were the neurotrophic factor *Bdnf*, the immediate early gene *Fos*, the proinflammatory cytokine *Interleukin 6* (*Il6*), and the oncostatin M receptor *Osmr* (type I cytokine receptor family). Both diet and genotype had a significant effect on genes involved in various physiological response, including inflammation and stress (*Il6ra*, *Nos1*, and *Npy*). A larger number of transcripts (neurotransmitter receptors, transcription factors, factors involved in neurodegenerative disorders, Table 2) exhibited a significant effect of genotype and, perhaps, more importantly, a significant interaction between the two variables. For example, transcripts exhibiting interactions between genotype and diet (Table 2 and Supplementary Figure 2) include *Adora2a*, an adenosine receptor involved in inflammation, and regulation of sleep, along with genes involved in metabolic and neurodegenerative diseases (*Adcy8, Lrrk2*), as well as in neuronal excitability and synaptic activity (*Calb2*, *Dlx1*). Strikingly, the expression of the sleep regulated gene *Homer1* was significantly influenced by the interaction between the two variables, and its levels were significantly increase in the WT-KD in comparison to the WT-ND and BACHD-KD. To reveal the enrichment of certain biological pathways, we used the Reactome Database in Rosalind.bio and reported in Table 3, only those terms reaching statistical significance at a FDR < 0.05 in at least one comparison as these could reflect a biological response to the KD. This analysis showed changes in a diverse set of gene categories/biological functions, such as oxidative stress, transcription factors, cell adhesion molecules and inflammation. No overlaps were observed, and strikingly, the comparison with the most enriched pathways was the BACHD ND vs. WT ND (Table 3). Hence, the KD altered the expression of a number of transcripts in the striatum, some of which (e.g., *Bdnf, Homer1, Il6ra, Lrrk2, Scnb*) implicated in neurodegenerative disorders, inflammation or sleep and could be potential targets for future work.

**TABLE 2:** Ketogenic diet impacts gene expression in the striatum of WT and BACHD male mice.

	WT		BACHD				
GOI	ND	KD	ND	KD	Diet	Genotype	Interaction
*Dlx1*	65 ± 15	65 ± 12	43 ± 9	81 ± 33*	***F*_(1, 20)_ = 5.325; *P* = 0.032**	*F*_(1, 20)_ = 0.144; *P* = 0.709	***F*_(1, 20)_ = 5.552; *P* = 0.029**
*Smyd1*	30 ± 9	15 ± 2**	28 ± 5	**27 ± 6^#^**	***F*_(1, 18)_ = 11.00; *P* = 0.004**	*F*_(1, 18)_ = 3.441; *P* = 0.080	***F*_(1, 18)_ = 7.907; *P* = 0.012**
*Bdnf*	80 ± 34	105 ± 35	50 ± 24	83 ± 27	***F*_(1, 17)_ = 4.695; *P* = 0.045**	*F*_(1, 17)_ = 3.825; *P* = 0.067	*F*_(1, 17)_ = 0.110; *P* = 0.744
*Fos*	190 ± 65	250 ± 72	116 ± 20	258 ± 107*	***F*_(1, 18)_ = 10.77; *P* = 0.004**	*F*_(1, 18)_ = 1.138; *P* = 0.300	*F*_(1, 18)_ = 1.728; *P* = 0.205
*Il6*	55 ± 24	79 ± 30	45 ± 15	69 ± 7	***F*_(1, 20)_ = 7.991; *P* = 0.010**	*F*_(1, 20)_ = 1.465; *P* = 0.240	*F*_(1, 20)_ = 8.6e-008; *P* = 0.999
*Kcnb1*	349 ± 99	412 ± 52	332 ± 53	386 ± 31	***F*_(1, 20)_ = 5.049; *P* = 0.036**	*F*_(1, 20)_ = 0.686; *P* = 0.417	*F*_(1, 20)_ = 0.030; *P* = 0.864
*Osmr*	31 ± 7	47 ± 8*	40 ± 7	47 ± 10	***F*_(1, 20)_ = 11.64; *P* = 0.003**	*F*_(1, 20)_ = 2.040; *P* = 0.169	*F*_(1, 20)_ = 1.764; *P* = 0.199
*Cxcr4*	34 ± 10	26 ± 6	39 ± 5	34 ± 3	***F*_(1, 20)_ = 6.252; *P* = 0.021**	***F*_(1, 20)_ = 6.321; *P* = 0.021**	*F*_(1, 20)_ = 0.125; *P* = 0.726
*Dlgap1*	131 ± 40	146 ± 35	83 ± 12	134 ± 25	***F*_(1, 16)_ = 5.895; *P* = 0.027**	***F*_(1, 16)_ = 4.857; *P* = 0.042**	*F*_(1, 16)_ = 1.732; *P* = 0.207
*Il6ra*	18 ± 2	21 ± 3	21 ± 5	29 ± 7^*,#^	***F*_(1, 20)_ = 9.145; *P* = 0.007**	***F*_(1, 20)_ = 9.334; *P* = 0.006**	*F*_(1, 20)_ = 1.757; *P* = 0.199
*Nos1*	140 ± 36	168 ± 36	99 ± 22	146 ± 18	***F*_(1, 19)_ = 9.747; *P* = 0.006**	***F*_(1, 19)_ = 6.899; *P* = 0.017**	*F*_(1, 19)_ = 0.623; *P* = 0.439
*Npy*	233 ± 118	398 ± 132*	176 ± 45	272 ± 36	***F*_(1, 19)_ = 11.86; *P* = 0.003**	***F*_(1, 19)_ = 5.799; *P* = 0.026**	*F*_(1, 19)_ = 0.827; *P* = 0.375
*Adora2a*	1134 ± 266	1422 ± 358	1115 ± 207	**885 ± 134^##^**	*F*_(1, 20)_ = 0.079; *P* = 0.781	***F*_(1, 20)_ = *7.135*; *P* = 0.015**	***F*_(1, 20)_ = 6.223; *P* = 0.021**
*Lrrk2*	904 ± 120	1098 ± 167*	857 ± 110	**723 ± 67^###^**	*F*_(1, 20)_ = 0.364; *P* = 0.553	***F*_(1, 20)_ = 18.03; *P* = 0.001**	***F*_(1, 20)_ = 10.92; *P* = 0.004**
*Ntng1*	702 ± 309	314 ± 146*	668 ± 81	**767 ± 125^##^**	*F*_(1, 16)_ = 3.007; *P* = 0.102	***F*_(1, 16)_ = 6.276; *P* = 0.023**	***F*_(1, 16)_ = 8.541; *P* = 0.010**
*Drd2*	789 ± 296	882 ± 185	705 ± 124	598 ± 75	*F*_(1, 20)_ = 0.007; *P* = 0.935	***F*_(1, 20)_ = 5.694; *P* = 0.027**	*F*_(1, 20)_ = 1.685; *P* = 0.209
*Ggt1*	28 ± 6	23 ± 4	31 ± 6	**33 ± 3^#^**	*F*_(1, 20)_ = 0.485; *P* = 0.494	***F*_(1, 20)_ = 9.976; *P* = 0.005**	*F***_(1, 20)_** = 2.923; *P* = 0.103
*Htr1a*	37 ± 8	35 ± 9	32 ± 7	27 ± 7	*F*_(1, 20)_ = 1.394; *P* = 0.252	***F*_(1, 20)_ = 4.408; *P* = 0.049**	*F*_(1, 20)_ = 0.159; *P* = 0.695
*Nfkbia*	180 ± 25	187 ± 29	201 ± 16	218 ± 18	*F*_(1, 19)_ = 1.756; *P* = 0.201	***F*_(1, 19)_ = 7.324; *P* = 0.014**	*F*_(1, 19)_ = 0.302; *P* = 0.589
*Ninj2*	42 ± 11	30 ± 6	49 ± 11	**47 ± 7^#^**	*F*_(1, 20)_ = 3.674 *P* = 0.069	***F*_(1, 20)_ = 10.84; *P* = 0.004**	*F*_(1, 20)_ = 1.522; *P* = 0.232
*Sncb*	235 ± 75	259 ± 87	154 ± 21	212 ± 33	*F*_(1, 20)_ = 2.725; *P* = 0.114	***F*_(1, 20)_ = 6.598; *P* = 0.018**	*F*_(1, 20)_ = 0.459; *P* = 0.505
*Acvrl1*	58 ± 9	92 ± 33	70 ± 18	59 ± 13	*F*_(1, 19)_ = 1.716; *P* = 0.206	*F*_(1, 19)_ = 1.258; *P* = 0.276	***F*_(1, 19)_ = 6.656; *P* = 0.018**
*Adcy8*	319 ± 126	208 ± 74	248 ± 20	327 ± 37	*F*_(1, 19)_ = 0.548; *P* = 0.468	*F*_(1, 19)_ = 0.237; *P* = 0.632	***F*_(1, 19)_ = 8.467; *P* = 0.009**
*Calb2*	240 ± 139	144 ± 79	158 ± 60	363 ± 158^*,#^	*F*_(1, 20)_ = 1.322; *P* = 0.264	*F*_(1, 20)_ = 2.092; *P* = 0.163	***F*_(1, 20)_ = 10.05; *P* = 0.005**
*Gata2*	28 ± 7	21 ± 7	24 ± 5	29 ± 3	*F*_(1, 20)_ = 0.229; *P* = 0.637	*F*_(1, 20)_ = 0.678; *P* = 0.419	***F*_(1, 20)_ = 5.545; *P* = 0.029**
*Homer1*	4964 ± 462	6080 ± 795*	5376 ± 635	**4614 ± 563^##^**	*F*_(1, 20)_ = 0.480; *P* = 0.496	*F*_(1, 20)_ = 4.252; *P* = 0.052	***F*_(1, 20)_ = 13.51; *P* = 0.001**

Two-way ANOVA followed by Holm-Sidak’s multiple comparisons test of selected genes identified by Nanostring transcriptomic analysis. A total of 30 differentially expressed genes was identified in 2 groups-comparisons with the highest number in the BACHD-KD vs. WT-KD comparison (Figure 3 and Supplementary Figure 2). Data are shown as the mean ± SD of 5–6 animals/genotype/diet regimen. Degrees of freedom are reported within parentheses, alpha = 0.05. Asterisks indicate significant difference within genotype (i.e., diet effect), whilst crosshatches those between genotypes (i.e., same diet). Bold type indicates statistical significance. ND, normal diet; KD, Ketogenic diet.

**TABLE 3:** Top enriched biological pathways extrapolated using the Reactome database (https://app.rosalind.bio/).

				WT KD vs. ND	BACHD KD vs. ND	ND BACHD vs. WT	KD BACHD vs. WT
						
Term ID	Term name	# Genes in term	# Genes in cluster	FDR-adjusted *P-*value	FDR-adjusted *P-*value	FDR-adjusted *P-*value	FDR-adjusted *P-*value
R-MMU-450341	Activation of the AP-1 family of transcription factors	10	1	**–**	0.0992	**0.0341**	**–**
R-MMU-9018519	Estrogen-dependent gene expression	118	1	**–**	0.1179	**0.0408**	**–**
R-MMU-2871796	FCERI mediated MAPK activation	29	1	**–**	0.0992	**0.0341**	**–**
R-MMU-1059683	Interleukin-6 signaling	9	2	–	**0.0026**	–	0.0866
R-MMU-112411	MAPK1 (ERK2) activation	8	2	–	**0.0026**	–	0.0866
R-MMU-110056	MAPK3 (ERK1) activation	9	2	–	**0.0026**	–	0.0866
R-MMU-6794361	Neurexins and neuroligins	31	1	–	–	**0.0341**	–
R-MMU-2559580	oxidative stress induced senescence	109	1	**–**	0.0992	**0.0341**	–

Only the terms with a FDR adjusted *p*-value of less than 0.05 in at least one group comparison are reported. *P*-value adjustments were performed using the Benjamini-Hochberg method of estimating false discovery rates (FDR) as reported in the ROSALIND ^®^ Nanostring Gene Expression Methods statement. Bold type indicates statistical significance. ND, normal diet; KD, ketogenic diet.

**FIGURE 4:**
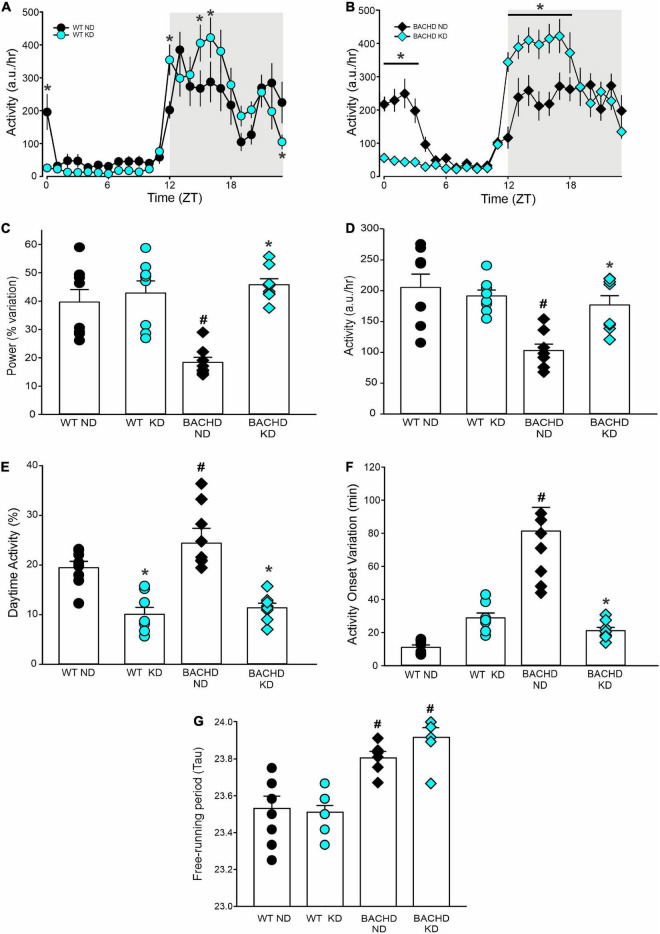
Ketogenic diet (KD) strongly improved the daily activity rhythms in male BACHD mice. The activity rhythms were monitored throughout the 3 months of exposure to the KD. The recordings of the last 3 weeks were analyzed and are shown (*n* = 8 mice per group). **(A,B)** Waveforms of daily rhythms in cage activity in 5–6 months old WT (circles) and BACHD (diamonds) mice under normal diet (ND; black) or KD (cyan). The gray shading indicates nighttime when mice were in the dark. The activity waveform (1 h bins) of each group was analyzed using a two-way ANOVA for repeated measures with treatment and time as factors. For WT mice, there were significant effects of time [*F*_(23_, _359)_ = 21.406, *P* < 0.001] but not of diet [*F*_(1_, _359)_ = 0.731, *P* = 0.393] along with a significant interaction of time and diet [*F*_(23_, _359)_ = 10.165, *P* < 0.001]. Similarly, for the BACHD mice, there were significant effects of time [*F*_(23_, _383)_ = 26.533, *P* < 0.001] but not diet [*F*_(1_, _383)_ = 3.768, *P* = 0.053]. Again, there was a significant interaction between the two variables [*F*_(23_, _383)_ = 17.329, *P* < 0.001]. *P < 0.05 differences between the 1 h bins, Holm-Sidak’s multiple comparisons test. **(C)** The KD restored the strength of the rhythms in the BACHD mice, while **(D)** increasing their activity per hour. **(E)** KD significantly decreased the improper activity during the light phase and **(F)** improved the activity onset. **(G)** The free-running circadian period (Tau) of the mice in constant darkness was measured. The BACHD mice exhibited a longer Tau that was not altered by the diet. Histograms show the means ± SEM with the values from the individual animals overlaid. Properties of the daily activity rhythms were analyzed using a two-way ANOVA with genotype and treatment as factors followed by the Holm-Sidak’s test for multiple comparisons (*n* = 8 per group) (see also Table 4). *P < 0.05 vs. mice on ND (effect of diet); ^#^*P* < 0.05 between genotypes (same diet).

BACHD mice exhibit altered sleep-wake cycles (34, 35). As shown in Figure 4, a number of parameters of the temporal pattern of cage activity (Figures 4A,B) were improved by the KD, in WT as well as mutants. Analysis of the activity waveform by two-way ANOVA with time and diet as factors indicated significant effects of time in both WT and BACHD mice, along with a significant interaction of the two variables. Most importantly, in the BACHD, the KD significantly reduced the non-characteristic activity during the day. Further analysis of the activity rhythms for all 4 groups (Table 4) found that the power of the rhythms (Figure 4C) was significantly altered by diet as was the average amount of activity per hour in the BACHD was increased by the KD (Figure 4D). The non-characteristic daytime activity as well as the variation in the onset of activity rhythms seen in the mutants were improved by the KD (Figures 4E,F). Finally, the lengthening of the free-running circadian period observed in the BACHD mice (Figure 4G) was not altered by the diet suggesting that the KD might not influence the molecular clock. Overall, the BACHD mutants exhibited weaker activity rhythms than WT, and KD improved many of the activity parameters.

**TABLE 4:** Impact of KD on activity rhythms and sleep.

	WT		BACHD				
	ND	KD	ND	KD	Diet	Genotype	Interaction
**Activity rhythms**							
Total activity (a.u./24 h)	2659 ± 1585	3648 ± 1180	4115 ± 812	4324 ± 982	*F*_(1, 30)_ = 0.05; *P* = 0.820	***F*_(1, 30)_ = 1.72; *P* = 0.02**	*F*_(1, 30)_ = 0.06; *P* = 0.800
Day activity (a.u./12 h)	651 ± 476	256 ± 58*	**1323 ± 204^#^**	472 ± 112*	***F***_(1, 30)_ **= 45.95; *P* < 0.001**	***F*_(1, 30)_ = 23.35; *P* = 0.03**	***F*_(1, 30)_ = 6.17; *P* = 0.019**
Day activity (%)	19.5 ± 3.7	10.1 ± 3.9	25.6 ± 6.4	11.4 ± 2.6	***F***_(1, 30)_ **= 58.72; *P* < 0.001**	***F*_(1, 30)_ = 5.86; *P* = 0.022**	*F*_(1, 30)_ = 2.53; *P* = 0.123
Night activity (a.u./12 h)	3009 ± 1255	3393 ± 1168	2792 ± 811	3853 ± 893	*F*_(1, 30)_ = 3.72; *P* = 0.064	*F*_(1, 30)_ = 0.11; *P* = 0.748	*F*_(1, 30)_ = 0.82; *P* = 0.374
Power (% variance)	39.7 ± 12.5	42.8 ± 12.0	**18.4 ± 5.0^#^**	45.8 ± 5.9*	***F***_(1, 30)_ **= 23.50; *P* < 0.001**	***F*_(1, 30)_ = 5.39; P = 0.03**	***F*_(1, 30)_ = 10.15; *P* = 0.004**
Onset variability (min)	11.0 ± 4.1	28.8 ± 8.3*	**81.4 ± 40.4^#^**	21.6 ± 5.6*	***F***_(1, 30)_ **= 8.23; *P* = 0.008**	***F*_(1, 30)_ = 17.96; *P* < 0.001**	***F*_(1, 30)_ = 27.90; *P* < 0.001**
**Sleep**							
Total sleep (min/24 h)	648 ± 74	667 ± 50	616 ± 20	**606 ± 51** ^#^	*F*_(1, 33)_ = 0.07; *P* = 0.791	***F*_(1, 33)_ = 6.14; *P* = 0.019**	*F*_(1, 33)_ = 0.62; *P* = 0.437
Day sleep (min)	454 ± 48	473 ± 13	444 ± 38	438 ± 37	*F*_(1, 33)_ = 0.40; *P* = 0.529	*F*_(1, 33)_ = 0.40; *P* = 0.529	*F*_(1, 33)_ = 2.61; *P* = 0.117
Night sleep (min)	194 ± 47	194 ± 43	**172 ± 35^#^**	167 ± 29	*F*_(1, 33)_ = 0.08; *P* = 0.768	***F*_(1, 33)_ = 4.38; *P* = 0.045**	*F*_(1, 33)_ = 0.41; *P* = 0.527
Fragmentation (# bouts)	10.7 ± 2.1	10.5 ± 1.7	12.8 ± 2.0	9.4 ± 1.6*	***F***_(_ **_1,33)_ = 5.93; *P* = 0.021**	*F***_(1, 33)_** = 0.04; *P* = 0.84	***F*_(1, 33)_ = 4.33; *P* = 0.046**
Sleep onset (ZT)	23.8 ± 0.5	0.4 ± 0.3	1.8 ± 1.4	0.2 ± 0.7	*F*_(1, 33)_ = 2.60; *P* = 0.118	***F***_(_ **_1,33)_ = 8.47; *P* = 0.007**	***F*_(1, 33)_ = 12.81; *P* = 0.001**

Comparisons of WT and BACHD mice fed a normal (ND) or a ketogenic diet (KD). Data were analyzed by two-way ANOVA using genotype and diet as factors followed by the Holm-Sidak’s multiple comparisons test and are expressed as the mean ± SD of 8 mice/genotype/diet regimen. Degrees of freedom are reported within parentheses, alpha = 0.05. Asterisks indicate significant difference within genotype (i.e., diet effect), and crosshatches those between genotypes (animals on same diet). Bold type indicates statistical significance. au, arbitrary units.

**FIGURE 5:**
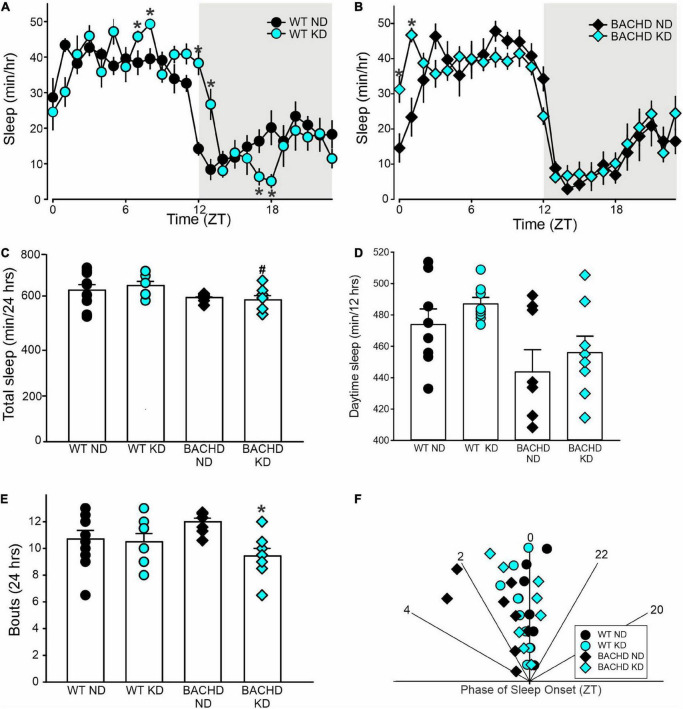
Modest improvement of the daily sleep behavior rhythms driven by the Ketogenic diet (KD) in BACHD mice. Sleep was measured in male WT and BACHD mice on normal diet (ND) or KD for 3+ months at 6–7 months of age (*n* = 8 mice per group). **(A,B)** Waveforms of daily rhythms in sleep behavior in WT (circles) and BACHD (diamonds) mice under ND (black) or KD (cyan). The gray shading indicates nighttime. BACHD mice on KD displayed an increase in sleep at ZT 0 and 1. The activity waveform (1 h bins) of each group was analyzed using a two-way ANOVA for repeated measures with treatment and time as factors. **(A)** For WT mice, there were significant effects of time [*F*_(23_, _431)_ = 30.483, *P* < 0.001] but not diet [*F*_(1_, _431)_ = 0.525, *P* = 0.469], as well as a significant interaction between the two variables [*F*_(23_, _431)_ = 4.149, *P* < 0.001]. **(B)** For the BACHD mice, there were significant effects of time [*F*_(23_, _383)_ = 32.808, *P* < 0.001] but not diet [*F*_(1_, _383)_ = 0.843, *P* = 0.359]. Again, there was a significant interaction of the two factors [*F*_(23_, _383)_ = 2.3619, *P* < 0.001]. **(C,D)** The KD did not affect total sleep or sleep in the day-time in the BACHD mice; still, **(E)** a significant decrease in the number of sleep bouts in the 24 h was observed. **(F)** KD reduced the phase delay in sleep onset shown by the BACHD on ND. Hence, the BACHD mice on KD were almost indistinguishable from WT. Histograms in **(C–E)** show the means ± SEM with the values from the individual animals overlaid. Properties of the daily sleep rhythms were analyzed using two-way ANOVA with genotype and treatment as factors followed by the Holm-Sidak’s test for multiple comparisons (*n* = 8 per group) (see also Table 4). *P < 0.05 vs. mice on ND (effect of diet); ^#^*P* < 0.05 between genotypes (same diet).

The amount of sleep is controlled by homeostatic mechanisms and was largely unaltered by the KD. For WT and BACHD mice, analysis of the sleep waveform (Figures 5A,B) indicated significant effects of time, but not diet, as well as a significant interaction between the factors. The total amount of sleep in a 24-h cycle was modestly reduced in both BACHD groups and unaltered by the diet (Figure 5C and Table 4). Overall, the KD did not significantly increase sleep behavior during the day (Figure 5D) despite the increase seen at ZT 0 and 1. The BACHD on KD displayed a reduced total number of sleep bouts in a 24-h period (Figure 5E and Table 4) as well as an advance in sleep onset (Figure 5F and Table 4). The mutant mice on ND started sleeping at ZT 1.8 ± 1.4 (108 min after lights on), while those on the KD at ZT 0.2 ± 0.7 or 12 min after lights-on. In summary, the KD produced a modest reduction in sleep fragmentation and corrected the phase delay in sleep onset seen in the BACHD mice.

**FIGURE 6:**
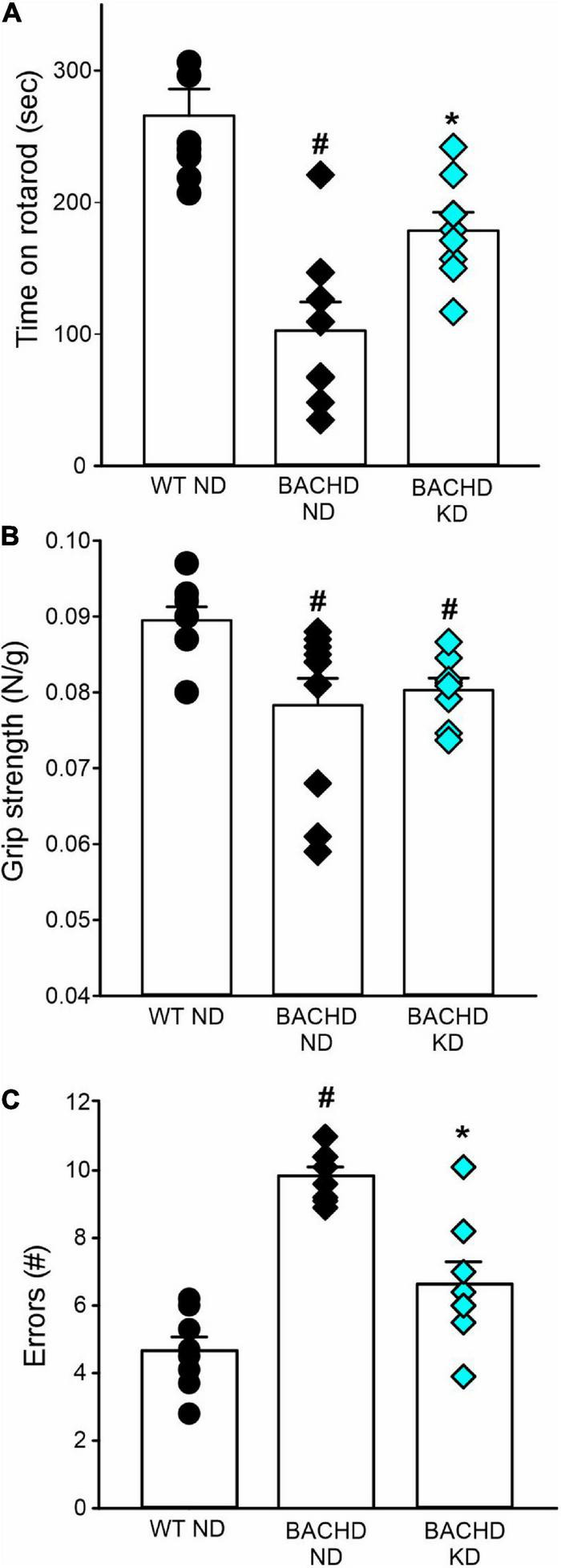
Ketogenic diet (KD) resulted in improved motor functions in the BACHD mice. Behavioral measures including time on rotarod, grip strength, and errors on the challenging beam were measured in WT and BACHD mice fed with either ND or KD at about 6.5–7 months of age (*n* = 8–10 mice per group) after monitoring sleep and activity rhythms. We have previously found that the BACHD exhibit deficits in all these tests and WT data are shown for comparison. **(A)** The time and performance on the rotarod were significantly improved in the BACHD on the KD. **(B)** Grip strength was not affected by the diet in the BACHD, that are not different by their counterpart on ND. **(C)** The total number of errors on the challenging beam test was significantly reduced in the BACHD mice on the KD. Histograms show the means ± SEM with the values from the individual animals overlaid. Motor data were analyzed using two-way ANOVA with genotype and treatment as factors followed by the Holm-Sidak’s test for multiple comparisons (see also Table 5). *P < 0.05 vs. mice on ND (effect of diet); ^#^*P* < 0.05 between genotypes (same diet).

The defining symptoms of HD are centered on motor dysfunction hence, we hypothesized that KD should also improve motor performance in the BACHD model. Motor performance was assessed using well-defined tests: the accelerating rotarod, grip strength, and challenging beam tests (Figure 6 and Table 5). As previously described, the BACHD mice exhibited worse performance in all three measures compared to WT at 6 months of age. The KD improved the performance of the mutant mice in the rotarod and challenging beam, but did not impact grip strength (Figure 6 and Table 5). The KD did not alter the performance of the WT mice in the rotarod or grip strength assays while the challenging beam was not evaluated. Overall, the improvement in motor performance exhibited by the BACHD on KD is a key finding of this study.

**TABLE 5:** Impact of Ketogenic diet on motor performance.

	WT		BACHD				
	ND	KD	ND	KD	Diet	Genotype	Interaction
Rotarod (s)	265.8 ± 57	276.8 ± 54	102.6 ± 62^#^	178 ± 40*	***F*_(1, 31)_ = 8.34; *P* = 0.007**	***F*_(1, 31)_ = 63.33; *P* < 0.001**	*F*_(1, 31)_ = 2.30; *P* = 0.140
Grip strength (N/g)	0.089 ± 0.005	0.087 ± 0.006	0.078 ± 0.004^#^	0.080 ± 0.002^#^	*F*_(1, 33)_ = 0.25; *P* = 0.622	***F*_(1, 33)_ = 6.62; *P* = 0.015**	*F*_(1, 33)_ = 0.03; *P* = 0.871
Errors on beam (#)	19.5 ± 3.7	NA	25.6 ± 6.4^#^	11.4 ± 2.6*	***F***_(_ **_1,23)_ = 58.72; *P* < 0.001**	***F*_(1, 23)_ = 5.86; *P* = 0.022**	*F*_(1, 23)_ = 2.53; *P* = 0.123

Grip strength measures for each animal were normalized to its body weight. Data were analyzed by two-way ANOVA with genotype and diet as factors followed by Holm-Sidak’s multiple comparisons test and are expressed as the mean ± SD of 8–10 mice/genotype/diet regimen. Degrees of freedom are reported within parentheses, alpha = 0.05. Asterisks indicate significant difference within genotype (i.e., diet effect), whilst crosshatches those between genotypes (i.e., same diet). Bold type indicates statistical significance. ND, normal diet; KD, ketogenic diet; NA, not available.

## Discussion

We have previously shown that scheduled feeding improves the sleep/wake cycle and motor performance in mouse models of HD (21, 22). These types of time-restricted feeding schedules produce a range of changes including an increase in ketosis (13). Hence, we became interested in the possibility that ketogenesis could underlie the improvements seen in these earlier works (36). However, HD patients and mouse models are known to exhibit a range of metabolic dysfunctions (6, 7) and so it was important to confirm that KD produced the intended effects in the BACHD model. We utilized a KD with moderately high protein, no sugars and predominantly healthy fats, quite distinct from that in some prior work: a “high-fat diet” with high sugar (averaging a stunning 20% of total Kcals from sucrose). Measuring body weight and composition, we found that the BACHD mice held on KD weighed less and exhibited lower body fat compared to the mutant mice held on ND (Figure 1). The mutant mice exhibited higher levels of βHB than WT on a normal diet and, importantly, the *ad libitum* KD generated a rhythm in ketone bodies peaking in the night (Figure 1). This is consistent with prior work by the Sassone-Corsi laboratory, who also found that KD-fed mice exhibit a robust oscillation in βHB serum levels (37). These rhythms are presumably driven by daily rhythm in feeding along with the well-characterized rhythm in the production of ketones in the liver (23, 38). These experimental observations provide a nice example of how a change in diet can generate a rhythm in a key metabolic parameter and thus fall into the category of chrono-nutrition.

### Microbiome

Prior work has provided evidence that the KD drives changes in the microbiome that can be measured in fecal samples (30, 39). Therefore, we sought to determine if the KD produced similar changes in the BACHD model. As measured by changes in species abundance, we confirmed that KD drove dramatic changes in the microbiome of both BACHD and WT mice (Figure 2 and Table 1). Changes in microbiota composition under KD could be due to either a maladaptive or an adaptive condition of the gut. Broadly, the patterns in the changes in species abundance looked similar between the two genotypes. It is noteworthy that the probiotic *Akkermansia municiphila* dramatically increased in abundance under KD. For example, under ND, this species represented 4% of the total species found in the BACHD while, under KD, this species increased to 44%. These observations are very similar to prior work showing that the KD can dramatically increase *A. muciniphila* (40, 41). Although not the focus of the present study, *A. muciniphila* is associated with improved metabolic health in both animal and human studies (42–44). This species could be an interesting focus of future experiments designed to determine if the changes in the microbiome impacted the behavioral outcomes. Interestingly, work in a *Drosophila* model of HD, suggest that the gut bacteria can regulate the pathology of HD (45), indicating that KD-driven changes in the microbiome could even influence HD-pathology. At a minimum, the results of our analysis confirm that the KD alters the composition of the gut microbiota in the BACHD model.

### Transcriptional changes in the striatum

Since one of the features of HD is transcriptional changes in the striatum (32, 33), we sought to determine if the KD could alter the transcriptional landscape in this brain region. Prior work found evidence that the KD increased the number of rhythmic genes in the liver and gut with a sharp peak in the middle of the day (37). While we did not examine gene expression in the striatum across the circadian cycle, we did not see much evidence for a large change in transcription in the striatum at night. However, there were a few transcripts altered by diet alone including *Bdnf*, *Fos*, *Il6*, and cytokine receptor *Osmr* (Figure 3, Supplementary Figure 1, and Table 2). BDNF is of particular importance in HD as a variety of studies have reported reduced levels in both patients and animal models (46–48), which is likely to have implications for neuronal survival. Prior studies have found evidence of a KD-driven increase in BDNF levels (49, 50) although these findings were not universal (51). A larger number of transcripts were altered by both genotype and diet and more importantly, several exhibited a significant interaction between these two factors including, among others Adora2a, *Nos1*, *Homer1*, and *Il6ra* (Figure 3, Supplementary Figure 1, and Table 2). Interestingly, neural activity in Nos1 expressing neurons is thought to be critical for sleep regulation (52, 53) and levels of *Homer1* change in response to sleep deprivation (54, 55). While exploring the possible function of these transcriptional changes is beyond the scope of the present study, hopefully these transcriptional changes can be pursued in future work. In the context of this study, the transcriptional analysis does demonstrate that diet can modulate gene expression in the striatum and presumably other regions within the nervous system. Overall, the data from both the microbiome and transcriptional analyses demonstrate that the KD is biologically impactful in the BACHD model.

### Sleep and activity

Disturbances in the sleep/wake cycle including prolonged latency to fall asleep, sleep fragmentation and difficulty maintaining wakefulness during the day are common in HD and often become apparent years before the onset of classic motor symptoms (56). Similarly, mouse models of HD also exhibit a disrupted circadian rest/activity cycle that mimics the symptoms observed in human patients (34, 57, 58). We have previously found that scheduled feeding (TRF) improved many of these deficits in animal models (21, 22). In the present study, we found that the KD enhanced several parameters of activity rhythms including rhythm power,% of activity in the day, and variation in activity onset such that the treated BACHD were indistinguishable from WT controls (Figure 4 and Table 4). One key exception is the lengthening of the free-running circadian period seen in the BACHD mice under constant dark conditions. The failure of the KD to correct this change suggests that the molecular circadian clock at the level of the suprachiasmatic nucleus (SCN) was unlikely to be altered by the diet. This finding is consistent with our prior work in which we found that TRF altered the phase of the PER2:LUC rhythms measured *in vivo* and *in vitro* outside of the SCN but did not impact the amplitude or phase of the rhythms measured in the SCN (21). In humans, there is also evidence that TRF alters rhythms in metabolites without perturbing clock gene expression (18).

In the present study, we found that the KD reduced the known sleep fragmentation and corrected the delay in sleep onset of the mutants (Figure 5 and Table 4). Since we were using a behavioral assay, we could not determine if the diet also impacted changes in the EEG power distribution, such as changes in beta and gamma activity, which are commonly seen in HD models (59–62). In humans, the published data on KD and sleep was inconsistent although at least one study did find that this regimen did increase slow wave sleep in men (63).

### Motor performance

HD is a movement disorder with hallmark symptoms of this disease including motor deficits and loss of neurons within the basal ganglia circuits. Importantly, KD did delay the reduction in motor performance as measured by rotarod and challenging beam (Figure 6 and Table 5). On the other hand, grip strength was not improved by KD. We have previously found improved motor performance on these same assays in the BACHD and Q175 lines of mice at the same age using scheduled feeding (21, 22). In the R6/2 mouse model, providing food only during the activity period (12 h feed/fast cycle) did improve performance of the mice on a battery of neurological tests (SHIRPA) as well as locomotor activity levels (64). The beneficial impact of KD on motor performance could be dependent upon or independent from the improvements in circadian output. In prior work, we found that the improved circadian behavior was correlated with improved motor function when we used a scheduled feeding protocol (22). This finding is at least consistent with the possibility that the improved sleep/wake cycle driven by the KD underlies the improved motor function in the treated mice. Thus, KD joins a growing list of interventions including TRF (21, 22); sleep-inducing drugs (65, 66), stimulants (67, 68), bright light & restricted wheel access (69) and blue light (28), which can improve motor function in HD models. This body of work supports our general hypothesis that circadian-based interventions and chrono-nutrition can improve symptoms in neurodegenerative disorders (70).

### Mechanisms

We do not know the mechanism through which the KD produced the benefits that we observed in the BACHD mice. Generally, the KD shifts the body into a state of elevated ketone body production and increasing serum ketone bodies, called ketogenesis or ketosis. Increasing the production of ketone bodies by the liver is dependent upon achieving a period during which glycogen stores are being depleted, such as during fasting, or by consuming a low-carbohydrate, high-fat diet, such as the one used in this study. During these states, ketone bodies are generated at higher levels, regulated by the rate limiting enzymes carnitine-palmitoyl transferase 1a (CPT1A), gating beta-oxidation, and hydroxymethyl-glutaryl CoA synthase 2 (HMGCS2), gating ketone body production, which are expressed in a circadian regulated manner (23). Notably, βHB and acetoacetate readily enter tissue and cross the blood–brain barrier through monocarboxylic transporters (38, 71). The primary fate of ketone bodies is to be converted to acetyl CoA in extrahepatic mitochondria and enter the Krebs cycle at the level of citrate, bypassing glycolysis to generate ATP. Notably, this happens with higher efficiency and lower production of ROS compared to glucose (72). While most of the βHB that is used as an energy source in the brain is synthesized by the liver, ketone bodies also undergo synthesis and release by astrocytes (73). Ketone bodies are thought to have direct effects on inflammatory molecules (74, 75), mitochondria (76), histone deacetylases (77), and BDNF expression (see above). Therefore, there are a number of important pathways through which the KD could be specifically benefiting the BACHD model as well as generally neurodegeneration (24, 25, 78).

While we do not know which biochemical pathways underlie the observed benefits of KD, there is growing evidence for sleep/wake regulation of the clearance of misfolded proteins in the brain glymphatic system, an astroglial-mediated interstitial fluid bulk flow (79). In HD and other neurodegenerative disorders, it has been proposed that sleep fragmentation drives a decline in clearance of brain waste. The activity of the glymphatic system is high during sleep and low during wakefulness (80). There are daily rhythms in both Aβ levels, as well as extracellular levels of tau (81). Sleep-deprivation increases Aβ plaque deposition, as well as tau pathology (82, 83). While sleep may be a direct driver, there is also good reason to suspect the circadian system involvement as well. For example, deletion of *Bmal1* causes severe circadian fragmentation, significantly blunts Aβ rhythms, and increases amyloid plaque deposition in a transgenic mouse model of AD (84). By improving sleep, the KD may be delaying the formation of aggregates in HD.

### Limitations

We were forced to bring this study to a premature closure because of COVID-19. Due to the research stoppage, there were some compromises, and this study presents several limitations. We did not collect data on the impact of the KD on the challenging beam task in WT mice. In addition, recently, concerns were raised on the negative impact of KD on cognition (85–87) and our study lacks cognitive measurements. Furthermore, the data on body composition and on the free-running period of circadian rhythms in locomotor activity were collected on a different cohort of mice. The study was originally designed to follow both sexes, as we have previously reported the presence of sex differences in the BACHD mice (35), with the females presenting less severe symptoms at early stages of disease. Sadly, another limitation of the present study is the usage of only male mice. Finally, we were unable to extend the study beyond 6 months of age, and age is likely another important critical factor. It should be underlined that we began the treatment at 3 months, when the BACHD young adults, largely, do not exhibit any phenotype at least for the parameters measured. In general, this line exhibits neuropathological changes such as striatal and cortical volume loss, protein aggregation and neuronal degeneration beyond 12 months (27). Intriguingly, KD was shown to reduce amyloid-β42 and β40 in APP (Amyloid Precursor Protein) mice (88), a mouse model for Alzheimer’s disease, and recently it has been suggested that nutritional ketosis could improve several astrocytic functions, while reducing astrogliosis (89). Future work will need to determine whether KD can ameliorate mitochondrial dysfunction, protein aggregation, ER stress, and/or facilitates autophagy later in disease progression. Obviously, because of all the above-mentioned limitations, more work is required, and caution should be used in the interpretation of our results.

## Summary

The weight of clinical and preclinical research indicate circadian and sleep dysfunction should be considered a core symptom of HD. We have previously shown that a feeding schedule benefits HD mouse models and that this treatment can drive a spike in ketones. Hence, in this study, we report that a KD effectively drove a rhythm in ketone bodies in serum of both WT and BACHD mice, but also dramatically altered their gut microbiome compositions and produced selective changes of the transcriptional landscape in the striatum. The KD strongly improved the activity rhythms as well as reducing fragmentation and the ameliorated the delayed sleep onset in the mutants. Motor performance on rotarod and challenging beam were also improved, while grip strength was unaltered. It is worth emphasizing that HD is a genetically caused disease with no known cure. Life-style changes that not only improve the quality of life but also delay disease progression for HD patients are greatly needed (90). Our study demonstrates the therapeutic potential of chrono-nutrition-based treatment strategies in a pre-clinical model of HD.

## Data availability statement

The data presented in this study are deposited in the Dryad repository, University of California Curation Center, accession number https://doi.org/10.5068/D1BT3K.

## Ethics statement

This animal study was reviewed and approved by UCLA Division of Animal Medicine.

## Author contributions

DW, GB, CG, and CC conceived the hypothesis and experimental design of this study. DW, RB, SV, SL, and TT performed the experiments. RB and DD’A analyzed the microbiome data. DW, TT, CG, and CC analyzed the data. DW wrote the first draft. CG and CC edited, wrote, and compiled the final version manuscript with contribution from the other authors. All authors contributed to the article and approved the submitted version.

## Footnotes

1
https://rosalind.bio/

2
https://app.rosalind.bio/

3
http://www.el-temps.com/principal.html

## Supplementary material

Supplementary Figure 1 Bar charts showing the fold changes in gene expression driven by the ketogenic diet in WT and BACHD animals and corrected *p*-values (app.rosalind.bio).

Supplementary Figure 2 Ketogenic diet driven changes in gene expression in the striatum of 6–7 months of WT and BACHD mice on ND or KD for 3+ months. Mice were kept on KD until euthanasia was performed at ZT14. Selected genes were analyzed by two-way ANOVA followed by Holm Sidak’s multiple comparisons test. The relative expression values obtained with the nSolver software were average (*n* = 5–6 animals per group) and are shown as the Mean ± SD. **P* < 0.05; ^**^*P* < 0.01 vs. mice on ND (effect of diet); ^#^*P* < 0.05; ^##^*P* < 0.01; ^###^*P* < 0.001 between genotypes (same diet) (see also Table 2).
