# Supplementary figures and images for: Dietary ketosis improves circadian dysfunction as well as motor symptoms in the BACHD mouse model of Huntington’s disease
Source: Front Nutr. 2022 Nov 3;9:1034743. doi: 10.3389/fnut.2022.1034743 (PMC9669764; doi:10.3389/fnut.2022.1034743)

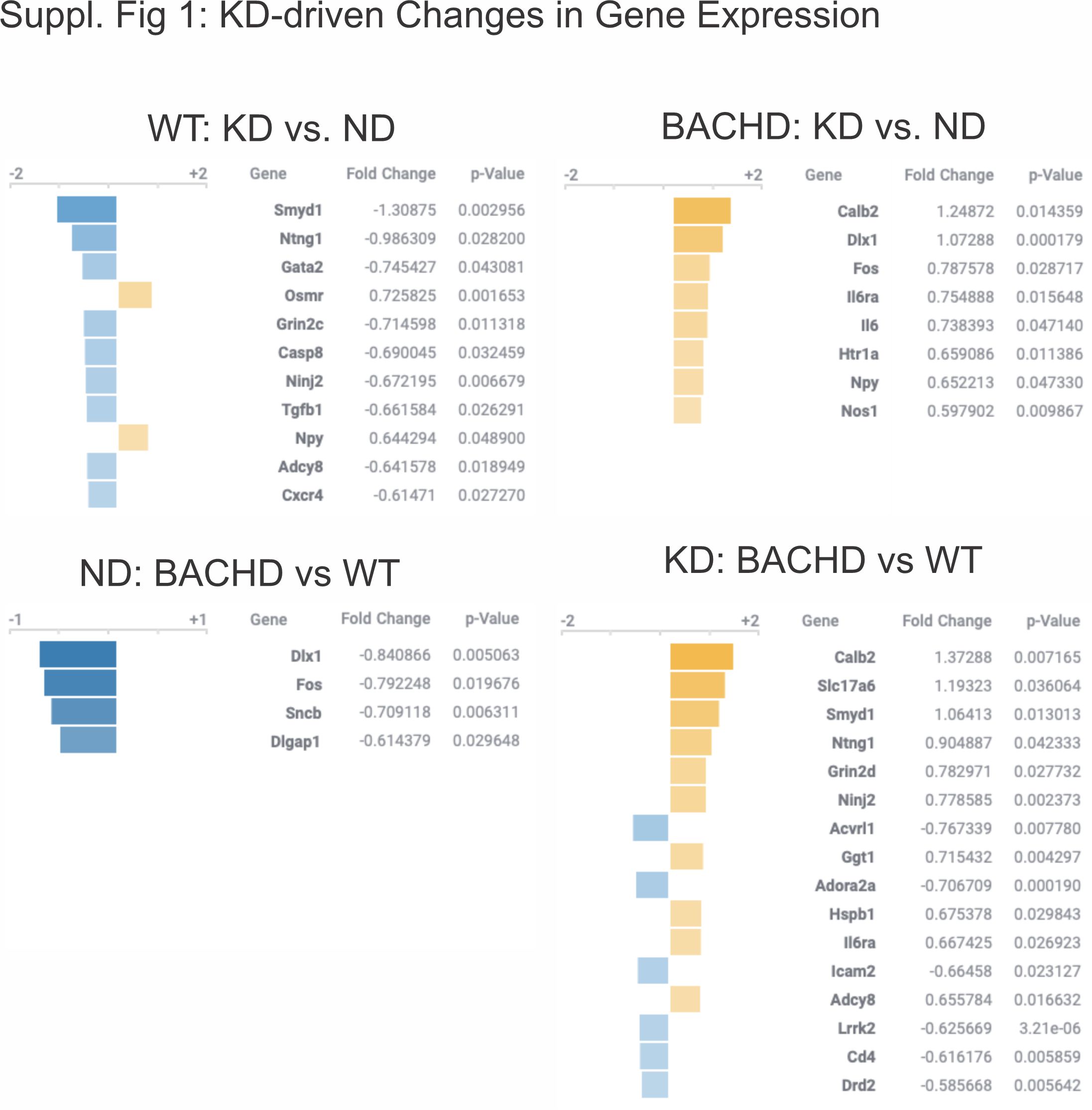

Supplement: Supplementary Figure 1 — Bar charts showing the fold changes in gene expression driven by the ketogenic diet in WT and BACHD animals and corrected p-values (app.rosalind.bio). [file Image_1.JPEG]

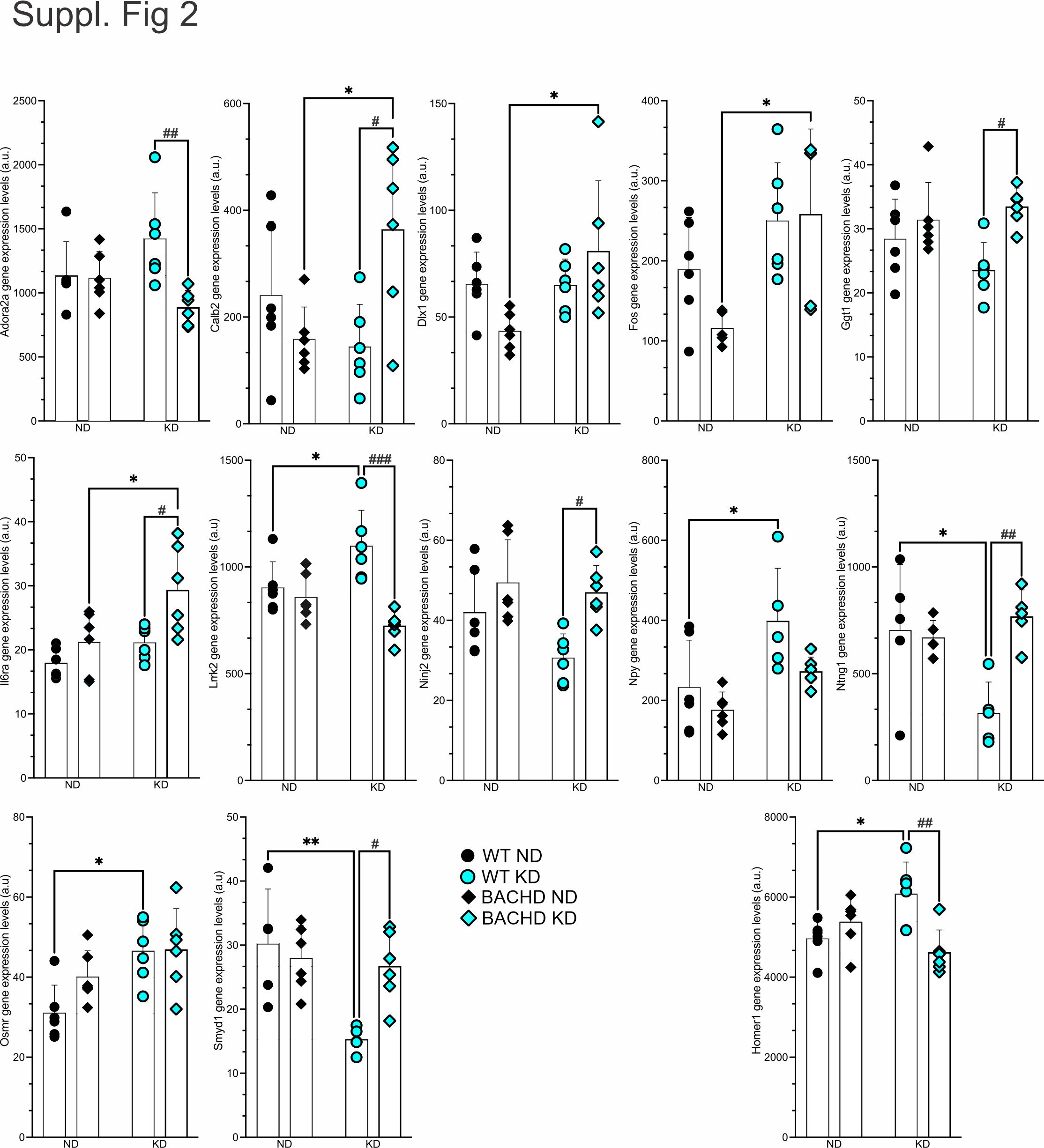

Supplement: Supplementary Figure 2 — Ketogenic diet driven changes in gene expression in the striatum of 6–7 months of WT and BACHD mice on ND or KD for 3+ months. Mice were kept on KD until euthanasia was performed at ZT14. Selected genes were analyzed by two-way ANOVA followed by Holm Sidak’s multiple comparisons test. The relative expression values obtained with the nSolver software were average (n = 5–6 animals per group) and are shown as the Mean ± SD. *P < 0.05; **P < 0.01 vs. mice on ND (effect of diet); #P < 0.05; ##P < 0.01; ###P < 0.001 between genotypes (same diet) (see also Table 2). [file Image_2.JPEG]
